# 

**Published:** 1895-12

**Authors:** 


### Books Received.

Osler: Practice of Medicine. D. Appleton & Co., New York.

Senn: Pathology and Surgical Treatment of Tumors. W. B. Saunders, Philadelphia.

American Text-Book of Obstetrics. Saunders, Philadelphia.

Moulin: Treatise on Surgery. Blakiston, Philadelphia.

Hyde & Montgomery: Manual of Syphilis and Venereal Diseases. Saunders, Philadelphia.

Pregnancy, Labor, and the Puerperal State. The F. A. Davis Co., Philadelphia.

Tyson: Practical Examination of the Urine. Blakiston, Philadelphia.

### INDEX TO VOLUME XXV.

PAGE.

Abnormality of the Genitals ... 134
Acne, Na ure and Treatment of—Bernard Wolff, M. D ... 400
Ammonal in Malarial Paroxysm ... 578
Abdominal Contusions, Element of Causation in—Thos. H. Manley, M. D ... 4, 65
Announcement ... 333, 380
Antitoxine, Death from Appendicitis—Floyd W. McRae, M. D ... 340
Appendicitis, Postcript to Report on—W. H. Halsted, M. D ... 28
Appendicitis—Chas. McBurney, M. D ... 32
Atlanta Medical Society, President's Address before—C. D. Hurt, M. D ... 113
Atlanta Medical Society ... 55
Atlanta Medical College ... 228
Albuminuria, a Book of Detachable Diet Lists for ... 227
Abrasions ... 59
Bronchitis ... 59
Bleaching Teeth ... 209
Bicycling for Women ... 429
Bandaging, a Manual of—C. H. Leonard, M. D ... 225
Bacteriologist, Laboratory Guide for the—L. Frothingham, M. D ... 227
Bladder, Passage of Hard, Non-fusible Phosphate of Lime from ... 252
Bath, The Warm, in Insomnia ... 426
Carcinoma of the Parturient Uterus, A Case of—George H. Noble, M. D ... 83
Cholecystotomy, in One and Two Sittings—J. McF. Gaston, M. D ... 123
Compound Fractures of the Skull, Treatment of, with Report of a Case—W. S. Goldsmith, M. D ... 130
Coal Tar Derivatives, The—J. W. Duncan, M. D ... 140
Congenital Cyanosis, A Case of—H. W. McLauthlin, M. D ... 199
Catarrhs and Colds, Home Treatment of—L. A. Dessar, M. D ... 225
College Graduates ... 276
Conjunctivitis, Granular ... 58
Chorea, Quinine in ... 208
Care of the Baby, The ... 384
Clitoridectomy ... 420
Collodion in Plastic Operations ... 425
Chloride of Lime in Snake Bite ... 427
Cranium, Remarks upon Trephining the ... 373
Cholera Infantum, Recent Therapeutics of ... 331
Dietetics, A Manual of ... 482
Digestion, Some New Theories of ... 417
Dose Book and Manual of Prescription Writing ... 227
Diseases of Women, A Text-Book of ... 43
Dysmenorrhœa ... 59
Duty of the State Medical Association, A ... 239
Deciduo-Sarcoma Uteri ... 224
Epidemic Cerebro-Spinal Meningitis, A Case of ... 80
Endometritis, A Contribution, etc—G. T. Harrison, M. D ... 495
Electrotherapy as a Means of Diagnosis in Gynæcology ... 517
Electric Current, General Therapeutic Effects of, etc ... 514
Empyema of the Antrum of Highmore, Treatment of ... 195
Embryology of the Eye, An Outline of the ... 225
Electricity, Ten Minutes in Medical—George S. Hull, Ph. G. M. D ... 350
Erysipelas, Ichthyol in ... 427
Eye, Wounds of the, and Their Treatment—T. E. Mitchell, M. D ... 291
Fractures of the Jaws—D. S. Arnold, D. D. S ... 181
Fistula in Ano ... 216
Facial Neuralgia, A Case of—D. S. Arnold, D. D. S ... 409
Fistulæ, Recto-vaginal, etc ... 16
Gem, A, Dedicated to that Rabelais of the Profession, T. C. M ... 143
Goitre, Treatment of—J. A. White, M. D ... 603
Genital Organs, An Abnormality of ... 134
Gynæcology, A Syllabus of ... 226
Gynæcological Fad, A ... 381
General Narcosis, A New Method of Inducing ... 379
Gynæcological and Obstetrical Society of Baltimore ... 321
Hæmorrhage After Tonsil Operations, Management of ... 245
Hernia, A Clinical Lecture on—W. B. de Garmo, M. D ... 361
Hernia, Complicated—S. E. Milliken, M. D ... 407
Hæmorrhoids; Prolapsed Rectum; New Operation—Merrill Ricketts, M. D ... 512
Hernia, A Case of Strangulated Femoral, etc ... 129
International Clinics ... 116
Ichthyol in Erysipelas ... 427
Insanity, A Case of, Cured by Removal of a Fibroid Tumor of the Ovary ... 355
Intermittent Fever, Treatment of ... 533
Infectious Disease, Immunity Conferred by an Attack of ... 423
Insomnia, Warm Bath in ... 426
Insanity, Increase of, and Some of its Causes ... 455
Influenza, Symptomatic Treatment of ... 272
Joint Diseases, Tubercular, in Children ... 1
Labor, Rupture of Uterus During Uring ... 210
Medical Association of Georgia ... 171, 255
Medical Meetings ... 279
Maryland Clinical Society ... 35, 165, 321
Medical Impertinence ... 214
Murphy Button, The Use of the ... 215
Multiple Neuritis, Case of ... 393
Medicine in Fiction ... 424

Medicine, Twentieth Century Practice of ... 435
Manual of Surgery, A ... 115
Medical Examiners for the State of Georgia ... 48
Medical Law, The ... 48
New York Letter ... 365, 410, 236, 467, 566
Newer Remedies, Notes on ...
New Method of Reducing General Narcosis ... 379
New Method of Making Palatable and Digestive Milk ... 379
Navel, The Care of the ... 471
Obstetric Practice, Some Rare Cases in ... 247
Osteo-Lipo-Chondroma ... 371
Opium Poisoning, Repeated Washings of the Stomach in ... 154
Prescriptions ... 58, 118, 174, 232, 288, 340, 390, 440, 492, 548, 590, 652
Pepto-Mangan for Ænemia, etc., The Use of ... 131
Peritoneal Adhesions of the Head of the Colon, etc., A Case of ... 128
Physiology, A Manual of Human—Raymond ... 42
Practical Analysis and Urinary Diagnosis—Purdy ... 45
Pemphigus, A Case of ... 502
Puerperal Infection of Urinary Tract, etc ... 78
Philadelphia Academy of Surgery ... 111, 584
Palmetto Wine ... 408
Puerperal Sepsis ... 421
Pruritus Ani ... 422
Perforating Typhoid Ulcer ... 22
Pyæmia and Septicæmia ... 445
Physicians and Life Insurance Companies ... 475
Polyuria, Due to Tubercular Peritonitis ...
Paralysis in Course of Pneumonia ... 274
Quinine in Chorea ... 208
Quinine, Acquired Idiosyncrasy for ... 94
Rheumatism and Its Successful Treatment ... 582
Richmond (Va.) Academy of Medicine and Surgery ... 104, 155, 220
Rectum, Prolapsed ... 512
Rectal Surgery, Some Points in ... 9
Rupture of Uterus During Labor ... 210
Ridley, Dr. F. M ... 279
Spectacle Glasses ... 298
Surgical Cases, Clinical Notes on ... 304
Spinal Caries, Complications Following ... 358
Spermatorrhœa, with Nocturnal Pollutions ... 378
Syllabus of Gynæcology ... 226
Strangulated Femoral Hernia ... 129
Skin Grafting, Thiersch's Method of ... 44
Syllabus of Lectures on Human Embryology ... 41
"Stammering of the Urinary Organs" ... 88
Salicylates in Acute Rheumatism ... 536
Tendon-Grafting ... 565
Tri-State Medical Association of Georgia, Alabama and Tennessee ... 570, 584
Tonsilitis, Acute Follicular ... 206
Treatment of Chancre ... 582
Transactions College of Physicians, Philadelphia ... 109
Treatment of Intermittent Fever ... 533
Tubercular Joint Disease in Children ... 1
Thiersch's Method of Skin Grafting ... 44
Typhoid Fever, Clinical Study in ... 558
Tumors, Necessity for Complete Extirpation of, etc ... 551
Thyroidectomy in Treatment of Goitre ... 144
Trional ... 208
Ten Minutes in Medical Electricity ... 350
Unjustifiable Operation, An ... 473
Use of the Murphy Button ... 215
Uterus, Rupture of, During Labor ... 216
Urethra, Unique Malformation of ... 219
Virginia Medical Society ... 520
Wounds of the Eye and Their Treatment ... 291
Women, Bicycling for ... 429
Woman and the Wheel ... 139

### AUTHORS AND CONTRIBUTORS.

Apostoli, Dr. G ... 514, 517
Arnold, D. S ... 181, 409
Allen, C. W ... 94
Abbe, Robert ... 22
Ashhurst, John, Jr ... 373
Bossi, L. M ... 210
Bacon, J. B ... 216
Bloomfield, U. J ... 219
Basinger, T. W ... 407
Barlow, W. Jarvis ... 80
Cerna, David ... 226
Calhoun, A. W ... 245
Duncan, J. W ... 140
Dunham, Theodore ... 44
Dacosta, J. C ... 115
Daland, Judson ... 116
De Garmo, W. B ... 361
Endmann, H ... 213
Edson, Cyrus ... 578
Fleming, Walter M ... 533
Farnham, A. B ... 206
Frothingham, L ... 227
Gaston, J. McF ... 123, 145
Gordon, W. S ... 252
Garrigues, Henry J ... 43
Goldsmith, W. S ... 130

Harris, H. F. .......... 393
Harrison, G. T. .......... 495
Halsted, W. S. .......... 28
Hurt, C. D. .......... 113
Halden, M. A. .......... 225
Hull, G. S. .......... 350
Harrington, A. F. .......... 128
Jones, Louis H. .......... 476
Lanphear, Emory .......... 355
Long, J. W. .......... 226
Levy, Robert .......... 195
Leonard C. Henri .......... 225
Milliken, S. E. .......... 1, 207, 358, 565
Manley, Thos. H. .......... 4, 65, 309
Mitchell, T. E. .......... 291
Mathews, J. M. .......... 9
McBurney, Chas. .......... 32
McRae, F. W. .......... 343
Manton, W. P. .......... 41
McLauthlin, H. W. .......... 199
Noble, G. H. .......... 83
Norris, R. C. .......... 72
Neilson, Thos. R. .......... 371
Ohmann-Dumesnil, A. H. .......... 508
Porter, Ira W. .......... 408
Purdy, C. W. .......... 42
Parsons, D. E. .......... 558
Patterson, A. B. .......... 298
Peyser, Mark W. .......... 220
Ridley, Frank M. .......... 239
Ricketts, Merrill .......... 512
Richardson, C. H. .......... 247
Raymond, J. A. .......... 41
Smith, S. E. .......... 510
Smith, A. Lapthorn .......... 16
Simes, J. Henry C. ..........
Taylor, W. J. .......... 109
Thomas, J. B. .......... 220
Thornton, E. Q. .......... 227
Wiber, D. D. .......... 209
Wile, W. C. .......... 578
Wiggin, F. H. .......... 551
Wolff, Bernard .......... 400

### The Southern Medical Record's Premium List.

Every physician sending in a subscription to **The Southern Medical Record** within 30 days, will be entitled to his choice of the following list of premiums:

1 Copy Leonard's Physician's Pocket Day Book.
1 Copy Leonard's Pocket Anatomist, Illustrated.
1 Copy Leonard's Materia Medica and Therapeutics.
1 Copy Leonard's Reference and Dose Book.
1 Copy Over 1,000 Prescriptions.
1 Copy Clark's Physical Diagnosis and Uranalysis.
1 Copy Owen's Post-Mortems, How to Make.
2 Pads Gynecological Tablets.
2 Pads Obstetric Tablets.
1 Copy Erasable Tablet Call Book.
1 Copy Fothergill's Brief Therapeutics.
1 Leonard's Flexible Uterine or Throat Applicator.
1 Hypodermic Syringe in Case.
1 Clinical Thermometer, warranted accurate.
1 Humerus (perforated metal) Splint.
4 Smith-Hodges Closed Lever Pessaries.
1 Sims' Uterine Sound.
1 Simpson's Uterine Sound.
1 Director and Tongue Tie, or Aneurism Needle.
1 Goodwillie's Nasal Speculum.
1 Hard Rubber Ear Syringe.
1 Set (four sizes) Gruber's or Toynbee's Ear Specula.
1 Dozen Surgeon's Needles, assorted sizes and kinds
10 Patent Eye-Threading Surgeon's Needles.
1 Dozen Red Gum Catheters.
1 Olive Pointed French Catheter.
1 Double-Current Catheter, male
1 Double-Current Catheter, female.
1 Double-Current Catheter, Lisle Thread.
1 Combined Male and Female Catheter.
3 Olive Point Lisle Catheters.
3 Olive Point Lisle Bougies.
1 Dozen Red Gum Bougies, assorted sizes.
1 Buttles Spear-pointed Uterine Scarificator.
12 Reels, assorted sizes, Surgeon's Silk.
1 Coil Silver Wire and Seven Reels Silk.
1 Dozen Carbolized Sponge Tents.
3 Coils Silver Suture Wire, different sizes.
1 Vial, three sizes, twisted silk.
1 Vial, three sizes, braided silk.
1 Vial Catgut (three sizes) Antiseptic Ligatures.
1 Tongue Depressor, folding fenestrated, nickel-plated.
1 Double Canula.
1 Nasal Speculum, Goodwillie's.
1 Posterior Nasal Syringe.
Any Single-bladed, shell handled pocket instrument.
1 Gross' Ear Scoop and Spoon Combined.
1 Artery Forceps, plated.
1 Dressing and Polypus Forceps, plated.
1 Pair Pocket Case Scissors, any shape.
1 Trocar, Exploring, Silver Canula.
1 Ashton's Glass Rectal Speculum.
1 Dieffenbach Self-closing Artery Clamp.
1 Soft Rubber Esmarch Bandage.
1 Gouley's Whalebone Guide.
2 Van Buren's Steel Sounds.
1 Set Phalangeal (three pieces) Splints.
1 Levis's Radial Splint.
1 Tibia and Fibula (perforated metal) Splint.
1 Maxilla Splint, perforated metal.
1 Femur Splint, perforated metal.
1 Long Patella Splint, perforated metal.
2 Throat Mirrors with handles.
1 Urinometer.
1 Fitche's Patent Pocket Scales.
1 Trocar (Hydrocele, etc.).
1 Ashton's Perineum Needle.
1 Exploring Needle, folding, shell handle.
1 Seaton Needle, folding, shell handle.
1 Lancet and Vaccine Comb combined.
1 Bristle Probang for Throat.
1 Otis Flexible Prostatic Guide.
1 Pair Retractors, Parker's or Mott's.
1 Metacarpal Saw, ebony handle
1 Nelaton's Probe, porcelain head.
1 Powder Insufflator, with patent scoop.
1 Wild's Angular Ear Forceps.
1 Dozen Sea-Tangle Tents.
¾ Dozen Sea-Tangle Hollow Tents.
1 Dozen Slippery Elm Tents.
1 DeVilbis Powder Blower.
1 Stylographic Pen.
1 Double Glass Magnifier.
1 Eye Scissors.
1 Dozen Surgeon's Sponges in Glass.
1 Three-blade Ivory Handle Pocketknife.
1 Pair Silver P. C. Probes.
4 Hypodermic Needles.
1 Thomas' Dull Curette.
1 Sponge Holder.
1 Bone Chisel, oval or flat.
1 Household Syringe, H. R. Tips.
4 Retroversion Pessaries.
4 Spiral Ring S. R. Pessaries.
2 Glass Vaginal Specula.
1 Reversible Trocar.
1 Fountain Pen.

